# The secreted protein S100A7 (psoriasin) is induced by telomere dysfunction in human keratinocytes independently of a DNA damage response and cell cycle regulators

**DOI:** 10.1186/2046-2395-3-8

**Published:** 2014-10-17

**Authors:** Alice de Castro, Fay Minty, Eva Hattinger, Ronald Wolf, Eric Kenneth Parkinson

**Affiliations:** 1 Centre for Clinical & Diagnostic Oral Sciences, Institute of Dentistry, Barts and the London School of Medicine and Dentistry, Queen Mary University of London, Turner Street, London E1 2AD, UK; 2 Department of Dermatology, Ludwig-Maximilian University Munich, Frauenlobstrasse 9-11, 80337 Munich, Germany; 3 Blizard Building, 4, Newark Street, London E1 2AT, UK

**Keywords:** Telomere, Senescence, Keratinocyte, Differentiation, SASP, S100

## Abstract

**Background:**

Replicative senescence is preceded by loss of repeat sequences of DNA from the telomeres that eventually leads to telomere dysfunction, the accumulation of irreparable DNA double strand breaks and a DNA damage response (DDR). However, we have previously reported that whilst telomere dysfunction in human keratinocytes is associated with a permanent cell cycle arrest, the DDR was very weak and transcriptional profiling also revealed several molecules normally associated with keratinocytes terminal differentiation, including S100A7 (psoriasin).

**Results:**

We show here that *S100A7* and the closely related *S100A15* (koebnerisin) are not induced by repairable or irreparable DSBs, ruling out the hypotheses that these genes are induced either by the low DDR observed or by non-specific cell cycle arrest. We next tested whether *S100A7* was induced by the cell cycle effectors *ARF* (p14^ARF^), *CDKN2A* (p16^INK4A^) and *TP53* (p53) and found that, although all induced a similar level of acute and permanent cell cycle arrest to telomere dysfunction, none induced *S100A7* (except p53 over-expression at high levels), showing that cell cycle arrest is not sufficient for its induction. The closely related transcript *S100A15* was also upregulated by telomere dysfunction, to a similar extent by p16^INK4A^ and p53 and to a lesser extent by p14^ARF^.

**Conclusions:**

Our results show that mere cell cycle arrest, the upregulation of senescence-associated cell cycle effectors and DNA damage are not sufficient for the induction of the *S100* transcripts; they further suggest that whilst the induction of *S100A15* expression is linked to both telomere-dependent and -independent senescence, *S100A7* expression is specifically associated with telomere-dependent senescence in normal keratinocytes. As both S100A7 and S100A15 are secreted proteins, they may find utility in the early detection of human keratinocyte telomere dysfunction and senescence.

## Background

Senescence is defined as a permanent cell cycle arrest that occurs following extensive cell divisions (replicative senescence, RS) or more acutely following a variety of cellular stresses (stress- or proliferation-induced senescence). In addition to engaging a permanent cell cycle arrest, senescent cells secrete a variety of proteins known as the senescence-associated secretory phenotype (SASP) or senescence-messaging-secretome [1,2]. These types of proteins are of extra interest because they may find a use in the non-invasive detection of senescent cells in ageing and other pathologies [3].

RS occurs following extensive rounds of cell division and is accompanied by the erosion of the chromosomal telomeres [4]. Telomeres are repeat sequences of DNA, TTAGGG and their associated proteins, which are known as the shelterin complex [5,6] and protect the DNA ends from being perceived by the cell as a DNA double strand break (DSB). Telomeres will shorten when cells divide in the absence of telomerase due to either the end replication problem [4,7,8], exonuclease digestion [9] or, in some circumstances, oxidative damage [10,11]. This culminates in the telomere being perceived as a DSB [12] and most senescent cells mount a significant DDR [13]. In addition, the G-rich telomere sequences are susceptible to oxidative damage and very inefficient at non-homologous end-joining repair leading to a preferential accumulation of irreparable DSBs (IrrDSBs) at the telomere [14,15]. Telomerase is deregulated in most human cancers including oral squamous cell carcinoma (OSCC [16]) and can immortalise normal and neoplastic keratinocytes that lack the expression of the *INK4A* locus or a combination of p53 and p16^INK4A^ proteins by lengthening the telomeres and restoring their function [17]. Telomerase has also been reported to remove the IrrDSBs at telomeres, but it is not clear whether this property is related to its canonical telomere-lengthening function [18].

Although keratinocyte stem cells are not thought to decrease in number during *in vivo* ageing [19], there is considerable evidence that they undergo an age-related loss of function (reviewed in [20]); as with increased chronological age, their progeny display increased levels of stochastic senescence *in vitro*[21] telomeric attrition [22-24] and an accumulation of p16^INK4A^[25,26], the last of which is reported to be inversely associated with longevity [26].

Telomere dysfunction and its associated DDR can be induced along with senescence by over-expressing a dominant-negative mutant of the shelterin protein telomere-repeat binding factor 2 (TRF2), TRF2 delta B delta M (*TRF2*^*ΔBΔM*^ or TRF2DN) [12]. However, although this manipulation induces permanent cell cycle arrest [27] and leads to underphosphorylated pRb (Minty and Parkinson, unpublished data), it does not generate a strong DDR in normal human keratinocytes, as exemplified by a lack of strong induction of p53 phosphorylation at serine 15, or 53BP1 foci and no detectable increase in p21^WAF^[28] or SMC1-phosphoS966 or Nbs1-phosphoS343 (Minty and Parkinson, unpublished data). In contrast, all of these DDR markers were induced by 8–16 gray of ionising radiation in a dose-dependent manner and p53 stabilisation by as low a dose as 1 gray ([28] and Minty and Parkinson, unpublished data). Similar results are seen when a neoplastic keratinocyte line, D17, lacking expression of p16^INK4A^ undergoes RS and telomere shortening [28]. Instead, in both situations, we observed an induction of several genes normally associated with keratinocyte terminal differentiation, including *HOPX* and *S100A7* and other genes not obviously related to differentiation (*ICEBERG* and the histone *HIST2H2BE*); in D17, we also observed a reduction in expression of all of these genes upon expression of telomerase in parallel with telomere lengthening, senescence bypass and an elimination of the very small increase in p53 phosphorylation at serine 15 [28]. As these genes showed potential as markers of telomere dysfunction, we investigated them further.

Our earlier work stimulated the hypothesis that signals other than DNA double strand breaks and the DDR could be generated from dysfunctional telomeres to increase the expression of proteins that might have diagnostic use. *S100A7* was of particular interest as its encoded protein is secreted by keratinocytes and is found in detectable amounts in human serum where it has found utility as a non-invasive marker of a subtype of lung cancer [29].

Alternatively, the small DDR observed in keratinocytes following telomere uncapping or shortening might be enough to induce the terminal differentiation genes. Indeed, DNA damage has been shown to contribute to tissue ageing by the induction of terminal differentiation [30]. To test this hypothesis, we examined the effects of both low- and high-dose ionising radiation [28,31] on the expression of the genes induced by telomere dysfunction and showed that *S100A7* was not increased in expression relative to the non-irradiated controls.

Additionally, the increased expression of keratinocyte differentiation genes could be a consequence of any form of senescence or growth arrest. To test this hypothesis and to investigate the role of the cell cycle proteins involved in senescence, we ectopically expressed *ARF (*p14^ARF^*)*, *CDKN2A (*p16^INK4a^*)* and *TP53* (p53) in human keratinocytes and showed that, despite similar levels of growth arrest, these manipulations did not induce the expression of *S100A7* (except for high levels of p53), although all three induced the expression of the *S100A15* (koebnerisin) gene, which is highly homologous to S100A7 (psoriasin) and difficult to discriminate when co-regulated. Their differential regulation by cell cycle inhibitors suggests that both S100 proteins have different functions that need to be further studied.

As the S100A7 protein was potentially a specific marker of telomere dysfunction, we investigated this further and showed it to be increased in normal keratinocytes following the induction of telomere dysfunction by the dominant-negative *TRF2* construct *TRF2*^*ΔBΔM*^.

## Results and discussion

### Markers of keratinocyte telomere dysfunction are not induced by DNA double strand breaks

In order to test whether the small DDR observed following keratinocyte telomere uncapping or shortening [28] was responsible for the induction of *HIST2H2BE*, *ICEBERG*, *HOPX* and *S100A7*, we exposed normal human keratinocytes to 2, 10 or 20 gray of ionising radiation (IR). All of these doses have been shown by ourselves ([28] and Minty and Parkinson, unpublished data) and others [31] to generate a strong DDR.

**Figure 1:**
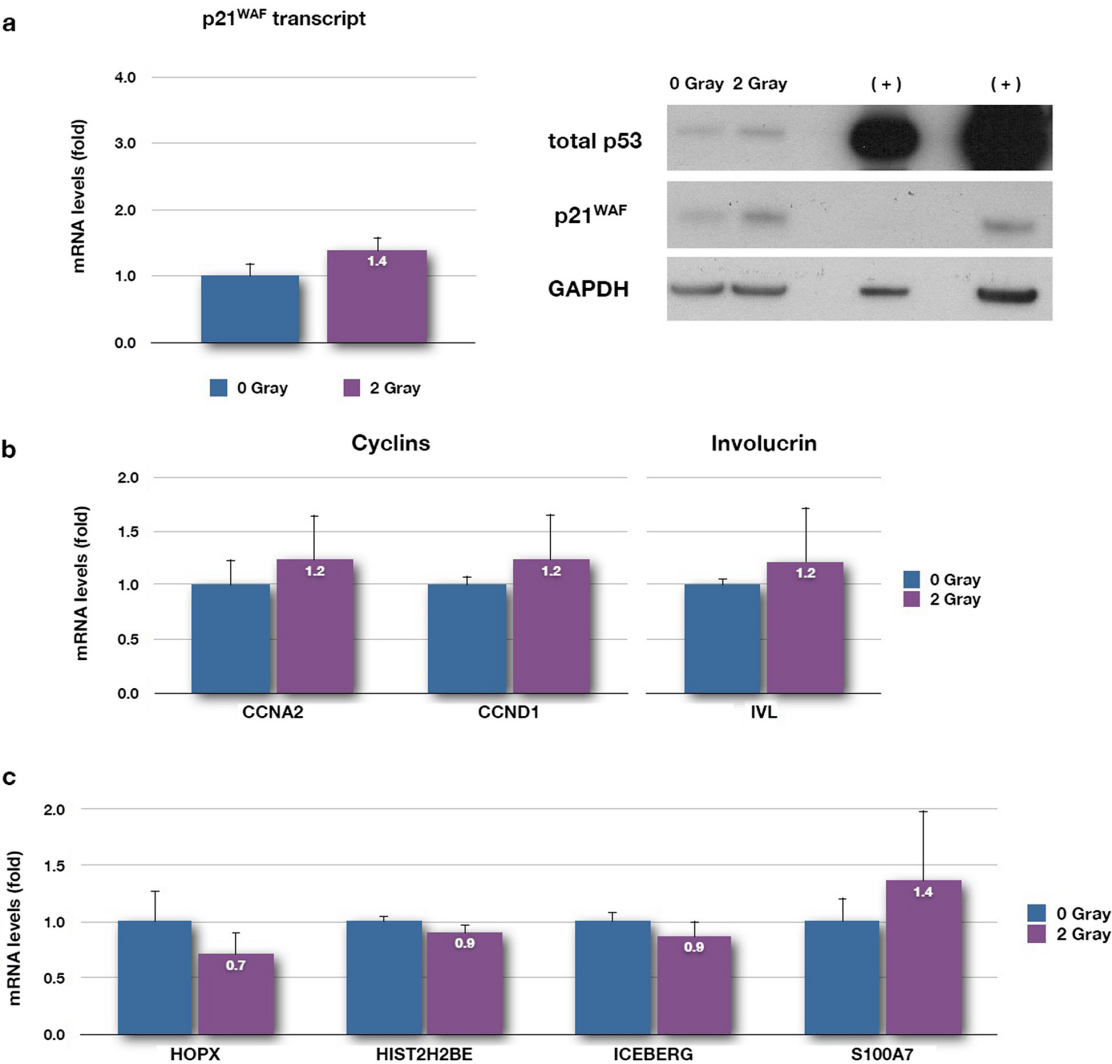
**Repairable levels of DNA damage do not replicate the effects of telomere uncapping in keratinocytes.** NHEKs were irradiated with 2 gray of IR and allowed 6 h to recover. Whole cell lysates were then prepared and analysed by reverse transcription quantitative PCR (RT-qPCR) and Western blotting for the indicated transcripts and proteins, respectively. **(a)***p21*^*WAF*^ transcript (graph), GAPDH, total p53 and p21^WAF^ proteins (blot). **(b)** Cyclin A2 (*CCNA2*), cyclin D1 (*CCND1*) and involucrin (*IVL*) transcripts. **(c)***HOPX*, *HIST2H2BE*, *ICEBERG* and *S100A7* (*S100A7*/*S100A15*) transcripts. Data are reported as a fold change in mRNA expression levels relative to the non-irradiated control (0 gray). Data are mean ± sd from three independent measurements (*n* = 3) in (b) and (c); in (a), only two out of the three independent measurements are represented. Legend: GAPDH, loading control; (+) symbol, positive control for total p53 and p21^WAF^ proteins (SVHFK cell line); last lane loaded with double the amount of total protein.

**Figure 2:**
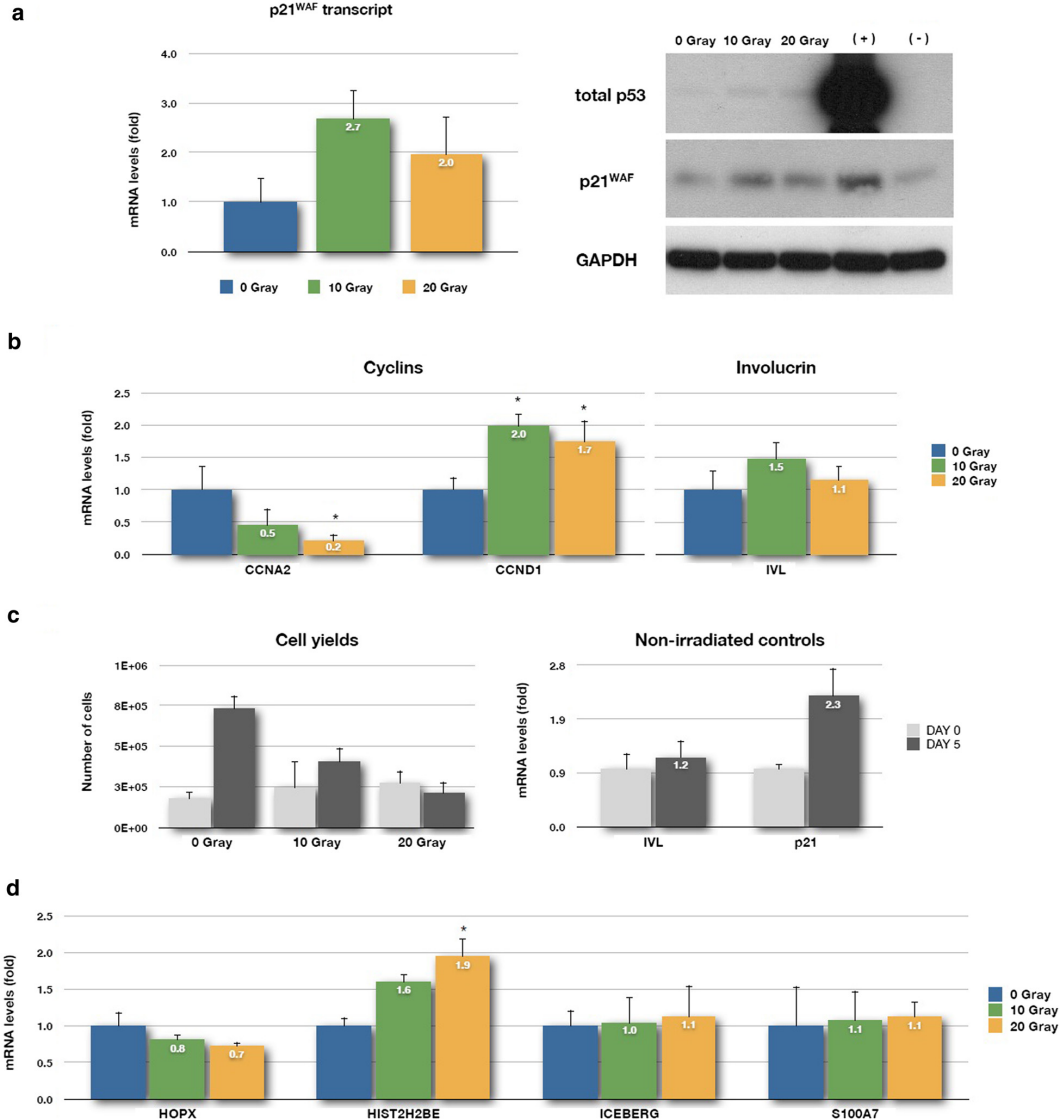
**Irreparable levels of DNA damage induce *HIST2H2BE *transcription but not *HOPX, ICEBERG *or *S100A7 *(*S100A7 */*S100A15 *)*.*** NHEKs were irradiated with 10 or 20 gray of IR and allowed 5 days to recover. Whole cell lysates were then prepared and analysed by RT-qPCR and Western blotting for the indicated transcripts and proteins, respectively. **(a)***p21*^*WAF*^ transcript (graph), GAPDH, total p53 and p21^WAF^ proteins (blot). **(b)** Cyclin A2 (*CCNA2*), cyclin D1 (*CCND1*) and involucrin (*IVL*) transcripts. **(c)** Total number of cells in all treated groups and *p21*^*WAF*^ and *IVL* transcripts in non-irradiated controls, before (day 0) and after (day 5) the recovery period. **(d)***HOPX*, *HIST2H2BE*, *ICEBERG* and *S100A7* (*S100A7*/*S100A15)* transcripts. Data are reported as a fold change in mRNA expression levels relative to the non-irradiated control (0 gray) in (a), (b) and (d); in (c), data are reported as a relative fold change in mRNA expression levels between day 0 (immediately after irradiation) and day 5 (after the recovery period). Data are mean ± sd from three independent measurements (*n* = 3) in (b), (c) and (d); in (a), only two out of the three independent measurements are represented. *Asterisks* (*p* <0.05) indicate significant change in transcript levels relative to non-irradiated cells and were calculated by one-way ANOVA followed by Tukey’s *post hoc* test. Legend: GAPDH, loading control; (+) symbol, positive control (SVHFK cell line); and (-) symbol, negative control (BICR-6 cell line) for total p53 and p21^WAF^ proteins.

The low dose of 2 gray was previously calculated to mimic the number of DNA double strand breaks generated by the transient uncapping all 92 telomeres by over-expressing *TRF2*^*ΔBΔM*^. The 10- and 20-gray doses induce senescence in all the keratinocytes in the population by introducing IrrDSBs. An ionising radiation dose of 2 gray induced p21^WAF^ transcript and protein, supporting previous data [28] that a significant DDR was generated (Figure 1a). However, the 2-gray dose did not reduce the levels of cyclin A2 and cyclin D1 transcript [32] (Figure 1b) as these transcripts take longer than 6 h to reduce and did not induce the early stages of terminal differentiation as assessed by involucrin transcript (Figure 1b). If anything, *HIST2H2BE*, *ICEBERG* and *HOPX* levels decreased slightly, and although *S100A7*/*S100A15* levels increased by 40%, this was not statistically significant (Figure 1c). Thus, the transient levels of DNA damage induced by telomere uncapping do not explain the previously reported induction of *HIST2H2BE*, *ICEBERG*, *HOPX* and *S100A7* transcripts [28], excluding this as a mechanism. However, as 2-gray irradiated cells were not growth arrested, let alone senescent, we tested the hypothesis that irreparable DNA double strand breaks, which are well known to cause senescence [31], could induce *HIST2H2BE*, *ICEBERG*, *HOPX* and *S100A7* transcripts. Figure 2a shows that 5 days following irradiation, p21^WAF^ transcript and both p21^WAF^ and p53 proteins were elevated in keratinocytes irradiated with both 10 and 20 gray and both doses of radiation reduced cyclin A2 transcript and induced cyclin D1 transcript (Figure 2b), both markers of senescence [32] and blocked keratinocyte multiplication (Figure 2c—cell yields). There was no evidence that IR-induced irreparable damage induced terminal differentiation as assessed by involucrin transcript levels (Figure 2b) or by increased colony size (data not shown), although p21^WAF^ expression was greater with increased colony size at day 5 (data not shown), consistent with the established association of p21^WAF^ with keratinocyte differentiation where its expression promotes the initial commitment of keratinocyte stem cell populations to differentiation [33] and contributes to differentiation-associated growth arrest [34]. Despite this, there was no observable induction of any of the genes of interest 5 days after IR, except *HIST2H2BE* (Figure 2d), which increased nearly twofold in both radiation doses and was significant at 20 gray. These experiments thus support the hypothesis that the irreparable DNA damage at the telomere induced by telomere uncapping could possibly be responsible for the induction of *HIST2H2BE*; although at this stage, non-specific growth arrest could not be ruled out. Nevertheless, these results reject the hypothesis that the low levels of DDR engendered by telomere uncapping were responsible for the induction of *ICEBERG*, *HOPX* and *S100A7*/*S100A15* expression. The results also argue against the hypothesis that these induced transcripts are the result of non-specific growth arrest, an assertion supported by the next set of experiments.

### The induction of permanent growth arrest by the cell cycle effectors *ARF*/p14^ARF^, *CDKN2A*/p16^INK4A^ and *TP53*/p53 does not replicate the effect of *TRF2*^***ΔBΔM***^ on the expression of *HIST2H2BE*, *ICEBERG*, *HOPX* and *S100A7*

**Figure 3:**
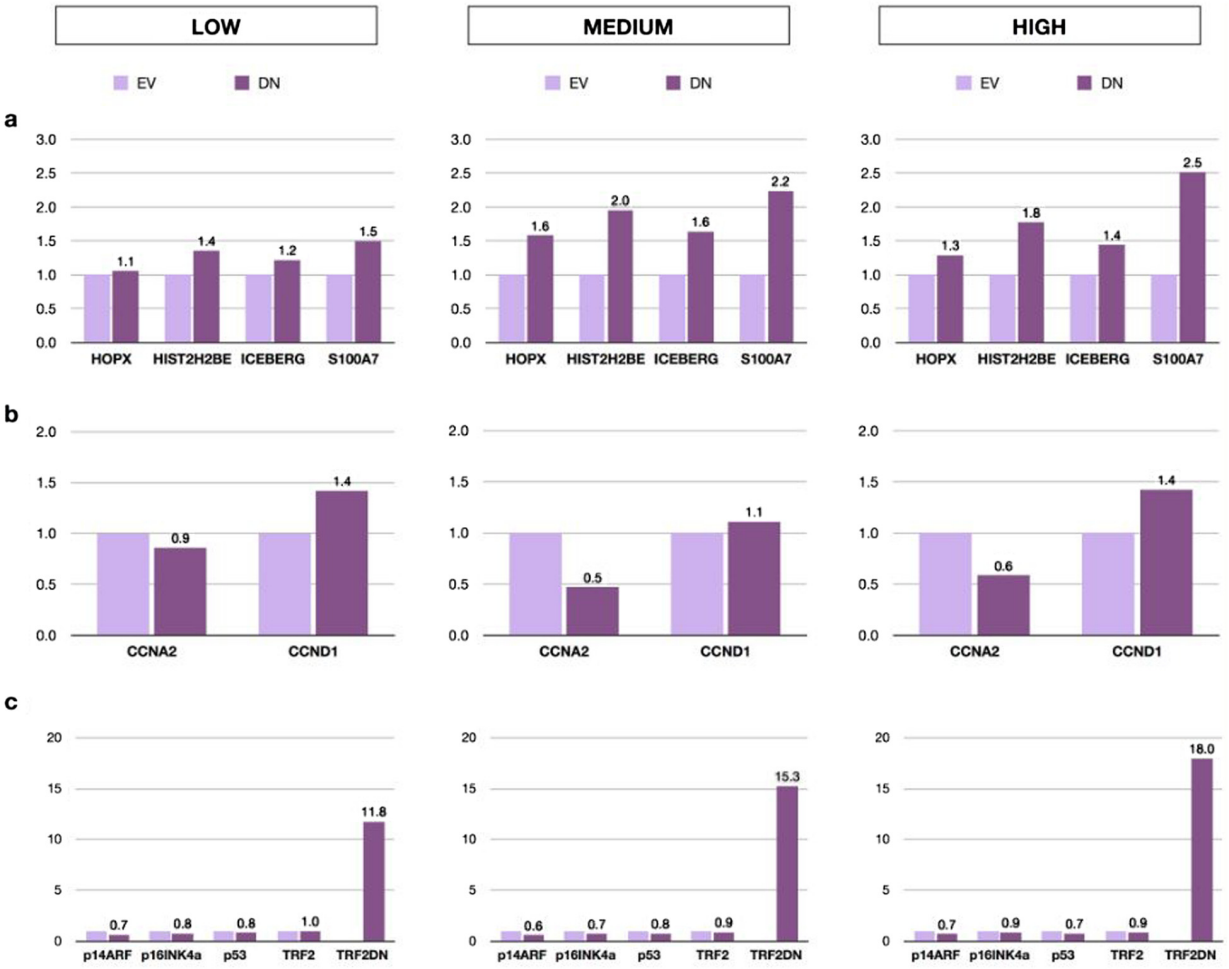
**Telomere dysfunction induces *HOPX*, *HIST2H2BE*, *ICEBERG *and *S100A7*/*S100A15 *but not the effectors of cell senescence.** NHEKs were transduced with TRF2^ΔBΔM^ in three independent experiments resulting in keratinocyte populations expressing TRF2^ΔBΔM^ at different levels: LOW (12-fold), MEDIUM (15-fold) and HIGH (18-fold). Cell extracts were analysed 5 days following expression of the transgene by RT-qPCR for induction of transcript levels of **(a)***HOPX*, *HIST2H2BE*, *ICEBERG* and *S100A7* (*S100A7*/*S100A15*); **(b)** Cyclin A2 (*CCNA2*) and Cyclin D1 (*CCND1*) and **(c)** effectors of senescence-associated cell cycle arrest *p14*^*ARF*^, *p16*^*INK4A*^ and *p53*. Endogenous TRF2 mRNA levels were also assessed to confirm they remain unaltered upon expression of its dominant-negative mutant TRF2^ΔBΔM^ (TRF2DN). Data are reported as a fold increase in mRNA expression levels relative to the respective empty vector (EV) control. Legend: EV, NHEK expressing empty vector control; DN, NHEK expressing TRF2^ΔBΔM^.

**Figure 4:**
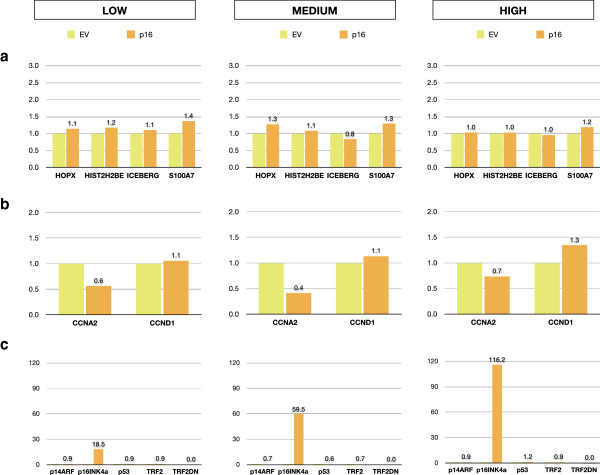
**Transcriptional profile of populations of normal human epidermal keratinocytes expressing p16**^**INK4A**^**.** NHEKs were transduced with p16^INK4A^ in three independent experiments resulting in keratinocyte populations expressing p16^INK4A^ at different levels: LOW (18-fold), MEDIUM (60-fold) and HIGH (116-fold). Cell extracts were analysed 5 days following expression of the transgene by RT-qPCR for induction of transcript levels of **(a)***HOPX*, *HIST2H2BE*, *ICEBERG* and *S100A7* (*S100A7*/*S100A15*); **(b)** Cyclin A2 (*CCNA2*) and Cyclin D1 (*CCND1*) and **(c)** effectors of senescence-associated cell cycle arrest *p14*^*ARF*^, *p16*^*INK4A*^ and *p53*. Data are reported as a fold increase in mRNA expression levels relative to the respective empty vector (EV) control. Legend: EV, NHEK expressing empty vector control; p16, NHEK expressing p16^INK4A^.

**Figure 5:**
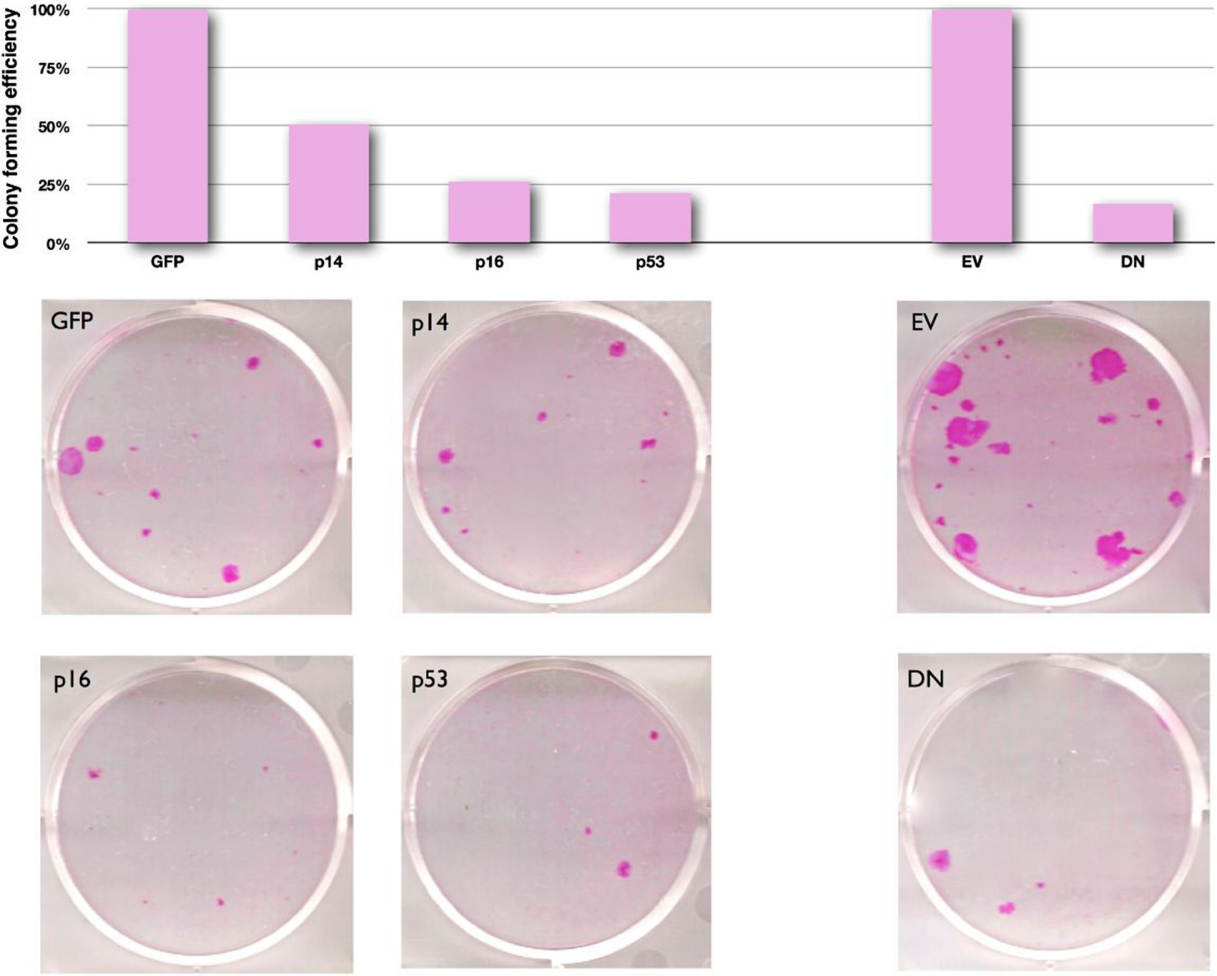
**Colony-forming efficiency analysis of NHEK transduced with *p14***^***ARF***^**, *p16***^***INK4A***^**, *p53 *and *TRF2***^***ΔBΔM***^**.** NHEKs were transduced with amphotropic retroviral particles using spinfection and, 48 h later, trypsinised and seeded at clonal density (7 × 10^3^ cells per 6-well plate). Cells were cultured for 2 weeks under drug selection and finally fixed and stained with Rhodamine B to reveal keratinocyte colonies. Colony-forming efficiency, displayed as percentage and relative to the respective EV control, was calculated by dividing the total number of colonies obtained per well by the total number of cells seeded per plate (7,000). Photos show wells representative of the results obtained for each construct. Legend: GFP, empty vector control for *p14*^*ARF*^, *p16*^*INK4A*^ and *p53*; EV, empty vector control for *TRF2*^*ΔBΔM*^ (DN). This is the result of a single experiment.

**Figure 6:**
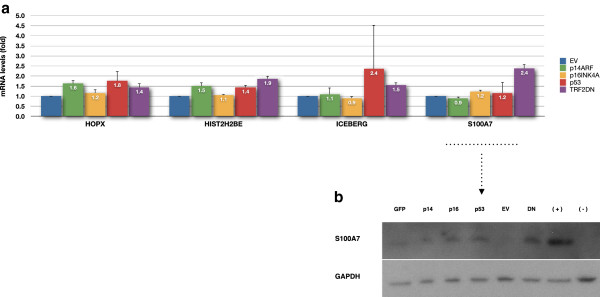
***HOPX*, *HIST2H2BE*, *ICEBERG *and *S100A7*/*S100A15 *transcripts in keratinocytes transduced with *p14***^***ARF***^**, *p16***^***INK4A***^**, *p53 *and *TRF2***^***ΔBΔM***^**. (a)** Candidate markers *HOPX*, *HIST2H2BE*, *ICEBERG* and *S100A7* (*S100A7*/*S100A15*) expression levels reported as a fold change in transcript relative to the respective empty vector (EV) control. Data are mean ± sd for two independent experiments (*n* = 2) corresponding to NHEK populations expressing MEDIUM and HIGH levels of the transgenes *p14*^*ARF*^, *p16*^*INK4A*^, *p53* and *TRF2*^*ΔBΔM*^. **(b)** Representative blot of S100A7 protein levels for the same NHEK populations. Legend: GFP, NHEK expressing pBabe-GFP empty vector; p14, NHEK expressing pBabe-p14^ARF^; p16, NHEK expressing pBabe-p16^INK4a^; p53, NHEK expressing pBabe-p53; EV, NHEK expressing pLPC empty vector; DN, NHEK expressing pLPC-TRF2^ΔBΔM^; (+) symbol, positive control (terminally differentiated NHEK); (-) symbol, negative control (telomerase-positive SCC-25 cell line).

In order to further test the specificity of *ICEBERG*, *HOPX* and *S100A7*/*S100A15* as markers of telomere dysfunction and to test the role of the downstream senescence effector proteins in their expression, we over-expressed p16^INK4A^, p14^ARF^ and p53 in normal human epidermal keratinocytes and compared them with the ectopic expression of *TRF2*^*ΔBΔM*^. Figure 3a shows the effects of telomere uncapping by the ectopic expression of *TRF2*^*ΔBΔM*^ on the expression of *HIST2H2BE*, *ICEBERG*, *HOPX* and *S100A7* confirming our earlier report that *TRF2*^*ΔBΔM*^ only induces expression of these genes at high levels of expression that cause a growth arrest illustrated by a 40%–50% reduction in *CCNA2* expression (Figure 3b). However, none of the classical cell cycle effectors of senescence-associated cell cycle arrest were affected at day 5 by telomere uncapping in human keratinocytes (Figure 3c). To test the effect of p14^ARF^, p16^INK4A^ and p53, we ectopically expressed these transgenes in human keratinocytes and compared their effects on *HIST2H2BE*, *ICEBERG*, *HOPX* and *S100A7*/*S100A15* transcripts with *TRF2*^*ΔBΔM*^. All four transgenes caused similar changes in both *CCNA2* and *CCND1* expressions within 5 days (Figures 3b, 4b and Additional file 1: Figure S1b and Additional file 2: Figure S2b) and similar levels of long-term growth arrest as shown by a 75% reduction in colony-forming efficiency in all groups except p14^ARF^, which caused only a 50% reduction in colony-forming efficiency (Figure 5). The forced expression of *TRF2*^*ΔBΔM*^ induced the expression of all four candidate markers as reported previously (Figure 4a) but did not induce the constitutive expression of any of the other transgenes tested within 5 days (Figure 4c). p16^INK4A^ did not induce the expression of *ICEBERG*, *HOPX* or *S100A7*/*S100A15* (Figures 4a and 6) showing that the induction of a permanent growth arrest in the absence of signals upstream of p16^INK4A^ was not enough to cause the increased expression of the candidate markers of telomere dysfunction-induced keratinocyte growth arrest. However, p53 did induce increased *ICEBERG* expression, although somewhat inconsistently (Figure 6a, Additional file 1: Figure S1a and Additional file 2: Figure S2a) and p53 and p14^ARF^ induced *HOPX* expression and to a lesser extent *HIST2H2BE* (Figure 6a, Additional file 2: Figure S2a), although there is no evidence that telomere uncapping induces high levels of p53 transcript (Figure 3c) or protein [28]. None of the transgenes induced the transcription of any of the other endogenous effectors of senescence, nor endogenous TRF2 (Figures 3c, 4c and Additional file 1: Figure S1c and Additional file 2: Figure S2c). No transgene, except *TRF2*^*ΔBΔM*^ (and high levels of p53), induced *S100A7* (Figure 6 transcript and protein), and for this reason, we pursued *S100A7*/*S100A15* as potential biomarkers of telomere dysfunction in the absence of DNA double strand breaks and studied these further.

### Distinct response of *S100A7 and S100A15* to telomere dysfunction and inducers of senescence

**Figure 7:**
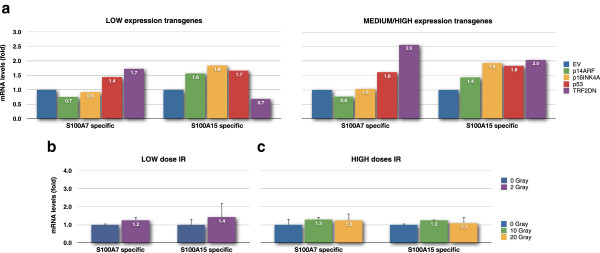
**Regulation of S100A7 (psoriasin) and S100A15 (koebnerisin) by TRF2**^**ΔBΔM**^**, the senescence effectors and IR.***S100A7* and *S100A15* gene expression levels **(a)** in keratinocytes transduced with *p14*^*ARF*^, *p16*^*INK4A*^, *p53* and *TRF2*^*ΔBΔM*^, reported as a fold change in transcript relative to the respective empty vector control; data are represented per NHEK population corresponding to LOW or MEDIUM/HIGH expression of the transgenes. **(b)** Keratinocytes irradiated with 2 gray (low dose IR) and **(c)** 10 or 20 gray (high doses IR), reported as a fold change in transcript relative to the non-irradiated control (0 gray); data are mean ± sd from three independent measurements (*n* =3). Legend: EV, empty vector; p14^ARF^, NHEK expressing pBabe-p14^ARF^; p16^INK4a^, NHEK expressing pBabe-p16^INK4a^; p53, NHEK expressing pBabe-p53; TRF2DN, NHEK expressing pLPC-TRF2^ΔBΔM^.

During the course of this study, the highly homologous *S100A15* (koebnerisin) was identified as part of the *S100A7*/*S100A15* gene subfamily [35] and it became evident that the primers we had used to detect *S100A7* also detected the closely related *S100A15* that, when expressed, shows a completely different expression pattern in squamous epithelia [35,36]. When specific primers were obtained and used to amplify each transcript separately, we observed that *S100A7* mRNA levels were in general about 100-fold higher than *S100A15* levels in keratinocytes (raw data not shown; presented data reported as a fold change relative to the relevant control) and their expression was distinct following induction of growth arrest by telomere uncapping and expression of the main senescence effectors. *S100A7* (psoriasin) was indeed induced by telomere dysfunction but not by p16^INK4A^, p14^ARF^ or p53 (induced by 1.6-fold with high levels of p53 but by 2.6-fold with high levels of *TRF2*^*ΔBΔM*^—Figure 7a). In contrast, *S100A15* (koebnerisin) was induced by the ectopic expression of p16^INK4A^ and p53 to a similar level to telomere uncapping and p14^ARF^ to a lesser extent (Figure 7a). Also, whilst *S100A15* was only induced by MEDIUM/HIGH expression levels of *TRF2*^*ΔBΔM*^, *S100A7* transcript was elevated even by a LOW expression of *TRF2*^*ΔBΔM*^, i.e. to levels incapable of engaging permanent growth arrest. Thus, *S100A7* seems to respond to even mild telomere dysfunction, which suggests not only better specificity than *S100A15* but also increased sensitivity to telomere damage. We also show that the elevation of either *S100A7* or *S100A15* was not merely a result of non-specific growth arrest since neither transcript was induced by repairable (Figure 7b) nor irreparable (Figure 7c) DNA double strand breaks. These results suggest that whilst the early increase in *S100A7* expression could be a specific consequence of telomere dysfunction in keratinocytes, the increase in *S100A15* expression may be a more general consequence of permanent growth arrest.

We previously reported that inducing telomere dysfunction in newborn human keratinocytes induced growth arrest and increased the expression of several genes, *HIST2H2BE*, *ICEBERG*, *HOPX* and *S100A7,* within 5 days, despite eliciting a very weak DDR that is typical of most cell types [28]. *HIST2H2BE*, *ICEBERG*, *HOPX* and *S100A7* were also expressed when a p16^INK4A^-deficient dysplasia line (D17) underwent replicative senescence and telomere shortening in the absence of a strong DDR, and these four transcripts were shown to be reduced upon the ectopic expression of the catalytic component of telomerase, telomere lengthening and elimination of the weak DDR [28].

The above experiments suggested that the four candidate genes *HIST2H2BE*, *ICEBERG*, *HOPX* and *S100A7* could be highly specific and early markers of keratinocyte telomere dysfunction, but several questions remained unanswered. For instance, it was possible that keratinocytes were unusually sensitive to the DDR, and that the weak DDR we observed was enough to induce the transcription of *HIST2H2BE*, *ICEBERG*, *HOPX* and *S100A7*. To test this, we subjected keratinocytes to both a low dose (2 gray) of γ irradiation that had been calculated to simulate the uncapping of 92 telomeres, and higher doses (10 or 20 gray) that had been reported to induce human cellular senescence and a strong DDR [28]. However, only *HIST2H2BE* was shown to be induced by γ irradiation. This is consistent with recent reports showing that chromatin components, such as H2A and H2B histones, are actively involved in the response to DNA damage [37,38]. *HIST2H2BE* is thus an active participant in the DDR and not a specific marker of telomere dysfunction-induced keratinocyte permanent growth arrest. In contrast, *ICEBERG*, *HOPX* and *S100A7* were not induced even by a strong DDR or indeed permanent growth arrest.

To further test the specificity of *ICEBERG*, *HOPX* and *S100A7* to telomere dysfunction, we induced permanent growth arrest in a different way: by the ectopic expression of the transcripts encoding the cell cycle inhibitors p14^ARF^, p16^INK4A^ and p53. This experiment had the dual purpose of testing the specificity of the above genes and also whether the cell cycle effectors were sufficient to induce them. The expression of p53 and p14^ARF^ did induce *ICEBERG* expression, although the effect of p53 was highly variable, and p53 induced *HOPX*. The result with p53 is a little difficult to reconcile with the absence of the induction of *ICEBERG* and *HOPX* following γ irradiation, but we have consistently failed to observe a large increase in total p53 protein in human keratinocytes following γ irradiation or telomere dysfunction and so the induction of *ICEBERG* and *HOPX* by p53 may be limited to situations such as UV exposure where p53 is stabilised and strongly over-expressed in human keratinocytes [39].

However, no transgene, except *TRF2*^*ΔBΔM*^, induced *S100A7* expression, and for this reason, we pursued *S100A7* as a potential early biomarker of telomere dysfunction in the absence of DNA double strand breaks. This result is also similar to that obtained for several SASP proteins, which are also not induced by the ectopic expression of the cell cycle regulator and effector of senescence, p16^INK4A^[40]. *S100A7* was over-expressed both at the transcript and protein level following telomere uncapping by ectopic expression of *TRF2*^*ΔBΔM*^.

TRF2 has been reported to be over-expressed in both basal and squamous cell carcinomas of the skin [41] and very recently has been shown to regulate the recruitment of natural killer cells by a cell extrinsic mechanism [42]. However, our earlier work showed that whilst the over-expression of *TRF2* at high levels did cause keratinocyte growth arrest it induced a distinct gene expression profile from *TRF2*^*ΔBΔM*^ that did not include *S100A7* (Minty et al., unpublished data). These data argue against our results being the consequence of *TRF2* over-expression as opposed to telomere uncapping.

During the course of the study, we became aware that the primers used to detect *S100A7* (psoriasin) also detected a closely related gene *S100A15* (koebnerisin). Koebnerisin, unlike psoriasin, is expressed in the epidermal basal layer and in cell types other than keratinocytes. However, when we used specific primers for *S100A7* and *S100A15* transcripts, we found that the major *S100* transcript specifically induced by telomere dysfunction was *S100A7* and that the expression of *S100A15* was very low. However, interestingly *S100A15* did seem to also be induced by the ectopic expression of p16^INK4A^ and p53; p16^INK4A^ is over-expressed in senescent keratinocytes *in vitro*[43] and increases in the epidermis of elderly humans [25,26] where it is also inversely associated with long-lived families [26] and it will be interesting to test whether *S100A15* follows a similar expression pattern to p16^INK4A^ in the epidermis of elderly subjects.

S100 proteins can participate in the squamous epithelial barrier as part of the cornified envelope, and S100A15 (koebnerisin) and S100A7 (psoriasin) both attracted interest because of their association with psoriasis. When released into the extracellular space, they synergise to participate in the innate immune response by acting as antimicrobial agents and by acting as chemoattractants for auxiliary immune cells and act as pro-inflammatory cytokines to amplify the immune response [44]. In normal skin, S100A7 (psoriasin) and S100A15 (koebnerisin) are both expressed by terminally differentiated keratinocytes of the upper epidermis, which are not replicative [36]. In contrast to psoriasin, koebnerisin is also produced by most cells in the basal epidermal layer, which might reflect the fact that most of the basal keratinocytes are non-dividing. Thus, koebnerisin could be a negative marker of replicating epidermal stem cells in the skin, which requires further investigation.

S100A7 (psoriasin) is upregulated in the early stages of tumour progression, where in its cytoplasmic form it has been reported to inhibit beta catenin signalling and act as a tumour suppressor in both breast epithelial cells [45] and keratinocytes [46]. S100A7 is downregulated when premalignant keratinocytes bypass senescence and is downregulated in the invasive parts of tumours *in vivo*. In contrast, in its secreted form, S100A7 binds to the receptor of advanced glycation endproducts (RAGE) on neighbouring tumour cells and can activate nuclear factor kappa B [47] and hence possibly members of the SASP [48] in neighbouring cells, including matrix metalloproteinases [49] and a variety of cytokines and other molecules [1]. Thus, S100A7 may mediate the effects of telomere dysfunction by first acting as a tumour suppressor intracellularly but may also act as a keratinocyte-specific SASP protein that can also spread its effects to neighbouring cells and as such may modulate to both ageing and tumour progression.

S100A7 protein is also detectable in human serum and as such might turn out to have potential as a non-invasive marker of keratinocyte telomere dysfunction in human ageing and disease. Indeed, serum S100A7 protein levels have been reported to be associated with squamous and large cell carcinomas of the lung in a cell type-specific manner [29]. The association of S100A7 protein with squamous cell carcinoma is something of a paradox as these cancers would very likely have deregulated telomerase [16] and reduced telomere dysfunction [50] in the immortal cells of the tumour. Furthermore, we have previously reported that the ectopic expression of telomerase in p16^INK4A^-deficient dysplastic keratinocytes can reverse the upregulation of *S100A7* following telomere attrition and senescence [28]; also, many telomerase-positive immortal keratinocyte lines have reduced levels of *S100A7* transcript (Hunter, Thurlow and Parkinson - unpublished data). However, significant levels of anaphase bridges and very short average telomere lengths have been reported in many human cancers, including squamous cell carcinomas [50,51], suggesting that considerable telomere dysfunction exists in portions of these telomerase-positive tumours.

At present, we can only speculate on the mechanism by which telomere uncapping upregulates S100A7, but recent data has suggested that the S100 proteins can be positively regulated by the histone demethylase JMJD3 in foetal human keratinocytes by repressing the H3K27me3 marks [52], and the histone demethylase LSD1 is required for estrogen-dependent S100A7 gene expression in human breast cancer cells [53]. Interestingly, telomere shortening and uncapping in telomerase-deficient mice also downregulates H3K27me3 and globally derepresses the genome [54], and therefore, the regulation of chromatin by telomere uncapping may offer a potential explanation for our results and the observation that telomerase can affect organismal ageing and healthspan when targeted only to epithelia as secreted proteins from cells with short telomeres could influence other organs in a paracrine manner [55].

## Conclusions

In summary, we have shown here that several gene transcripts (*ICEBERG*, *HOPX*, *S100A7* and *S100A15*) are upregulated following telomere uncapping in human keratinocytes independently of the DDR, and in the case of *S100A7*, this was also independent of cell cycle effectors such as p16^INK4A^, suggesting that the secreted form of S100A7 is a keratinocyte-specific SASP with the potential to non-invasively detect keratinocytes with dysfunctional telomeres.

## Methods

### Cell culture

Normal human epidermal keratinocytes, strain NHEK-131 (GIBCO-Invitrogen, Paisley, UK), were derived from a pool of a minimum of three neonatal foreskins and obtained at 6.8 mean population doublings (MPDs). Keratinocytes were cultured at 37°C in a 10% CO_2_/90% air with lethally irradiated 3T3 feeder cells in flavin-adenine enriched medium (FAD^-^). FAD^-^ consists of 3 parts DMEM 4.5 g/L glucose (Lonza, Slough, UK), 1 part Ham’s F12 (Lonza), 10% (*v*/*v*) Hyclone Fetalclone II serum (Fisher Scientific, Loughborough, UK), 20 mM HEPES buffer (Lonza), 100 U/ml penicillin, 100 U/ml streptomycin (Lonza) and 2 mM L-Glutamine (Lonza), supplemented with 1.8 × 10^-4^ M Adenine (Sigma-Aldrich, Poole, Dorset, UK), 5 μg/ml insulin (Sigma-Aldrich), 5 μg/ml transferrin (Sigma-Aldrich), 0.4 μg/ml hydrocortisone (Sigma-Aldrich) and 8.4 ng/ml cholera toxin (Fisher Scientific, Loughborough, UK). Medium was replenished every third or fourth day with FAD^+^ complete medium, which consists of FAD^-^ supplemented with 10 ng/ml of epidermal growth factor (Sigma-Aldrich).

### Irradiation

Cells were irradiated using a GSR D1 Cs-137 low dose-rate gamma irradiator (GSM, Leipzig, Germany) at 1.493 gray/min for 1 min 20 s for a total dose of 2 gray with a recovery period of 6 h or at 1.493 gray/min for 13 min 23 s for a total dose of 20 gray and 0.747 gray/min for 13 min 23 s for a total dose of 10 gray, both with a recovery period of 5 days.

### Retroviral transduction

Retroviral vectors pLPC-N MYC (12540 Addgene, Cambridge, MA.) and pLPC-NMYC TRF2^ΔBΔM^ (16069 Addgene) were donated by Titia de Lange (Rockefeller University, NYC, USA); pBABE-puro p14^ARF^, pBABE-puro p16^INK4a^ and pBABE-puro p53 were donated by Gordon Peters (London Research Institute, CRUK, London, UK); and pBABE-puro GFP was donated by Cleo Bishop (Blizard Institute, QMUL, London, UK). Vector DNA was amplified in XL1-Blue Competent Cells (Stratagene, La Jolla, CA, USA) and purified with the Plasmid DNA purification Maxi kit (Qiagen, Manchester, UK) according to manufacturer’s instructions. Retroviral supernatants were produced by transfecting plasmid DNA into Phoenix A packaging cells (Nolan Labs, Stanford, USA) using FuGENE®6 transfection reagent (Roche) at a 1 μg DNA to 2.5 μl FuGENE®6 ratio. Next, amphotropic supernatants were used to infect keratinocytes (after removing feeders) with two consecutive rounds of centrifugation at 300 rpm for 1 h at 32°C (spinfection), 6 h apart, in the presence of 5 ug/ml polybrene (Sigma-Aldrich) to facilitate viral uptake. Retroviral supernatant was replaced with FAD medium and transduced keratinocytes were kept under normal culture conditions in the presence of irradiated 3T3 ‘feeders’. Mock-transduced plates were treated with polybrene only. Puromycin (Sigma-Aldrich) selection (1 μg/ml) was introduced 24 h later and kept for 72 h following the 24-h period allowed for gene expression at which point the mock plates were dead. Selective medium was then replaced with FAD overnight and cell pellets collected by centrifugation.

### qPCR

Extraction of total RNA was performed using the RNeasy Mini Kit (Qiagen) according to manufacturer’s instructions, including sample homogenisation with QIAshredder (Qiagen) and DNase treatment with the RNase-free DNase Set (Qiagen). Purified RNA was reverse transcribed using with Finnzymes DyNAmo™ cDNA Synthesis Kit (New England BioLabs, Hitchin, UK) and the cDNA stored at -20°C. RT quantitative real-time PCR (absolute copy number quantification using standard curve for each gene) was performed using SYBR® green I Master (hot-start Taq polymerase master mix) in the LightCycler 480® qPCR system (Roche Applied Science, Welwyn Garden City, Herts., UK). Target gene relative expression levels were calculated in relation to keratinocyte reference genes *POLR2A* and *YAP1*[56] using the LightCycler 480® Relative Quantification Software (with built-in multiple reference genes normalisation algorithm).

Primers used were, CCNA2 fwd CCATACCTCAAGTATTTGCCATC and CCNA2 rev TCCAGTCTTTCGTATTAATGATTCAG for *CCNA2* cyclin A2 [GenBank: NM_001237.3]; CCND1 fwd CGTGGCCTCTAAGATGAAGG and CCND1 rev GTGTTCAATGAAATCGTGCG for *CCND1* cyclin D1 [GenBank: NM_053056.2]; HIST2H2BE fwd GGTAGATCCACCCTTATGCTT and HIST2H2BE rev TTAAGAGGGGAACACCATGAG for *HIST2H2BE* histone cluster 2, H2be [GenBank: NM_003528.2]; HOPX fwd ACTTCAACAAGGTCGACAAGC and HOPX rev GGGTCTCCTCCTCGGAAA for *HOPX* HOP homeobox [GenBank: NM_139212.3; GenBank: NM_032495.5; GenBank: NM_001145460.1; GenBank: NM_001145459.1; GenBank: NM_139211.4]; ICEBERG fwd CTTGCTGGATTGCCTATTAGAG and ICEBERG rev TTGAGGGTCTTCTTCACAGAG for *CARD18* caspase recruitment domain family, member 18 [GenBank: NM_021571.2]; IVL fwd TGCCTGAGCAAGAATGTGAG and IVL rev TTCCTCATGCTGTTCCCAGT for *IVL* involucrin [GenBank: NM_005547.2]; p14^ARF^ fwd CTACTGAGGAGCCAGCGTCTA and p14^ARF^ rev CTGCCCATCATCATGACCT for *CDKN2A* cyclin-dependent kinase inhibitor 2A, variant 4 or beta [GenBank: NM_058195.2]; p16^INK4A^ fwd CCAACGCACCGAATAGTTACG and p16^INK4A^ rev GCGCTGCCCATCATCATG for *CDKN2A* cyclin-dependent kinase inhibitor 2A, variant 1 or alpha [GenBank: NM_000077.3]; p21^WAF1^ fwd TCACTGTCTTGTACCCTTGTGC and p21^WAF1^ rev GGCGTTTGGAGTGGTAGAAA for *CDKN1A* cyclin-dependent kinase inhibitor 1A [GenBank: NM_000389.3; GenBank: NM_078467.1]; p53 fwd AGGCCTTGGAACTCAAGGAT and p53 rev CCCTTTTTGGACTTCAGGTG for *TP53* tumour protein p53 [GenBank: NM_000546.4; GenBank: NM_001126114.4; GenBank: NM_001126113.1; GenBank: NM_001126112.1; GenBank: NM_001126115.1; GenBank: NM_001126116.1; GenBank: NM_001126117.1]; POLR2A fwd GCAAATTCACCAAGAGAGACG and POLR2A rev CACGTCGACAGGAACATCAG for *POLR2A* polymerase (RNA) II (DNA directed) polypeptide A [GenBank: NM_000937.3]; S100A7 fwd AGACGTGATGACAAGATTGAC and S100A7 rev TGTCTTTTTTCTCAAAGACGTC for *S100A7* S100 calcium-binding protein A7 [GenBank: NM_002963.3]; S100A15 fwd ACGTCACTCCTGTCTCTCTTTACT and S100A15 rev TGATGAATCAACCCATTTCCTGGG for *S100A7A* S100 calcium-binding protein A7A [GenBank: NM_176823.3]; and S100A7/S100A15 fwd AAAGCAAAGATGAGCAACAC and S100A7/S100A15 rev AAGTTCTCCTTCATCATCGTC for co-amplification of both; TRF2 fwd CCAGATGAAGACAGTACAACCAA and TRF2 rev CCAGTTTCCTTCCCCATATTT for *TERF2* telomeric repeat binding factor 2 [GenBank: NM_005652.2]; TRF2DN fwd GTTGATTTCTGAAGAAGATTTGTT and TRF2DN rev GTGGAAGTAGAACTTGAGCAC for *TRF2*^*ΔBΔM*^; YAP1 fwd CCCAGATGAACGTCACAGC and YAP1 rev GATTCTCTGGTTCATGGCTGA for *YAP1* Yes-associated protein 1 [GenBank: NM_000389.3; GenBank: NM_078467.1]. *S100A7*/*S100A15* primers [28] amplify highly homologous transcripts for *S100A7* (psoriasin) and *S100A15* (koebnerisin). *S100A7*-specific and *S100A15*-specific primers were designed to independently quantify each transcript [57]. *HOPX* primers were designed to amplify all five transcript variants described for this gene. The design of a specific primer set for amplification of exogenous *TRF2*^*ΔBΔM*^ was performed manually, the forward sequence directed at the myc tag (located upstream of the gene insert) and the reverse sequence targeting the *TRF2*^*ΔBΔM*^ transgene in the retroviral construct. We have validated that all primer pairs are highly specific for the expected product without cross-amplification. All qPCR results were repeated as three complete experiments with two to three replicates each unless otherwise stated.

### Western blotting

Cells were lysed with M-PER® Mammalian Protein Extraction Reagent (Thermo Scientific) and cOmplete Mini EDTA-free protease inhibitor cocktail tablets (Roche). Mixture was gently shaken for 10 min at room temperature and cell debris removed by centrifugation at 14,000 g for 15 min. Supernatant was collected and kept on ice before being stored at -80°C. Protein concentration in the cell lysates was measured using the DC™ Protein Assay (BioRad, Hertfordshire, UK). Protein samples for Western blotting were prepared by adding 1x NuPAGE® LDS Sample Buffer (Life Technologies) followed by denaturation for 5 min at 100°C. Proteins were loaded onto NuPAGE® 10% Bis-Tris precast resolving gels (Invitrogen) and separated by gel electrophoresis at 130 V on 1x NuPAGE® running buffer (Invitrogen). Transfer to Immobilon™ PVDF membranes (Millipore, Watford, UK) was performed on transfer buffer (25 mM Tris, 190 mM glycine and 20% methanol) at 30 V for 90 min at 4°C. Membranes were blocked in 5% non-fat milk in Tris-buffered saline/Tween® 20 (TBS-T: 1 M Tris, pH 8.0; 5 M NaCl; 0.1% Tween® 20) for 1 h at room temperature. Next, membranes were probed with primary antibodies in 5% non-fat milk in TBS-T overnight at 4°C followed by incubation with HRP-conjugated secondary antibodies, prepared in 5% non-fat milk in TBS-T, for 1 h at room temperature. Immunodetection was performed with Amersham™ ECL Plus chemiluminescent detection system (GE Healthcare Life Sciences) and visualised on Amersham™ ECL Hyperfilm (GE Healthcare Life Sciences, Chalfont St. Giles, Bucks, UK). Primary antibodies used were mouse monoclonal anti-human p21^WAF1^ (C70) at 1:250 dilution (610233 BD Transduction Labs), mouse monoclonal anti-human p53 (DO-1) at 1:250 dilution (sc-126 Santa Cruz Biotechnology, CA), mouse monoclonal anti-human psoriasin/HID5/S100A7 at 1:1,000 dilution (IMG-409A Imgenex, San Diego, CA) and rabbit polyclonal anti-human GAPDH at 1:1,000 dilution (ab9485 Abcam Cambridge, UK). Secondary antibodies used were polyclonal goat anti-mouse IgG HRP-conjugated at 1:2,500 dilution (Fisher Scientific) and polyclonal goat anti-rabbit IgG HRP-conjugated at 1:2,500 dilution (Fisher Scientific). 

In the UK commercially derived human cells are not subject to further ethical approval by the purchaser and once passaged they are regarded as a cell line and exempt.

## Abbreviations

DDR: DNA damage response; DMEM: Dulbecco’s modified Eagle’s medium; DSB: DNA double strand break; FAD: flavin-adenine enriched medium; GAPDH: glyceraldehyde-3-phosphate dehydrogenase; HRP: horseradish peroxidase; irrDSB: irreparable DNA double strand break; MPD: mean population doubling; NHEK: normal human epidermal keratinocyte; OSCC: oral squamous cell carcinoma; RS: replicative senescence; SASP: senescence-associated secretory phenotype; TRF2 or TERF2: telomeric repeat binding factor 2; TRF2DN or TRF2^ΔBΔM^: dominant-negative TRF2 or TRF2 delta B delta M.

## Competing interests

The authors declare that they have no competing interests.

## Authors’ contributions

AC: study design, study execution, data preparation and writing of the manuscript. FM, EH, RW and EKP: study design and writing of the manuscript. All authors read and approved the final manuscript.

## Supplementary Material

Additional file 1: Figure S1 Transcriptional profile of populations of normal human epidermal keratinocytes expressing p14^ARF^. NHEKs were transduced with p14^ARF^ in three independent experiments resulting in keratinocyte populations expressing p14^ARF^ at different levels: LOW (89-fold), MEDIUM (142-fold) and HIGH (220-fold). Cell extracts were analysed 5 days following expression of the transgene by RT-qPCR for induction of transcript levels of (a) *HOPX*, *HIST2H2BE*, *ICEBERG* and *S100A7* (*S100A7*/*S100A15*); (b) Cyclin A2 (*CCNA2*) and Cyclin D1 (*CCND1*) and (c) effectors of senescence-associated cell cycle arrest *p14*^*ARF*^, *p16*^*INK4A*^ and *p53*. Data are reported as fold increase in mRNA expression levels relative to the respective empty vector (EV) control. Legend: EV, NHEK expressing empty vector control; p14, NHEK expressing p14^ARF^. Click here for file

Additional file 2: Figure S2 Transcriptional profile of populations of normal human epidermal keratinocytes expressing p53. NHEKs were transduced with p53 in three independent experiments resulting in keratinocyte populations expressing p53 at different levels: LOW (10-fold), MEDIUM (7-fold) and HIGH (29-fold). Cell extracts were analysed 5 days following expression of the transgene by RT-qPCR for induction of transcript levels of (a) *HOPX*, *HIST2H2BE*, *ICEBERG* and *S100A7* (*S100A7*/*S100A15*); (b) Cyclin A2 (*CCNA2*) and Cyclin D1 (*CCND1*) and (c) effectors of senescence-associated cell cycle arrest *p14*^*ARF*^, *p16*^*INK4A*^ and *p53*. Data are reported as fold increase in mRNA expression levels relative to the respective empty vector (EV) control. Legend: EV, NHEK expressing empty vector control; p53, NHEK expressing p53. Click here for file
