# Supplementary figures and images for: The secreted protein S100A7 (psoriasin) is induced by telomere dysfunction in human keratinocytes independently of a DNA damage response and cell cycle regulators
Source: Longev Healthspan. 2014 Oct 17;3:8. doi: 10.1186/2046-2395-3-8 (PMC4304136; doi:10.1186/2046-2395-3-8)

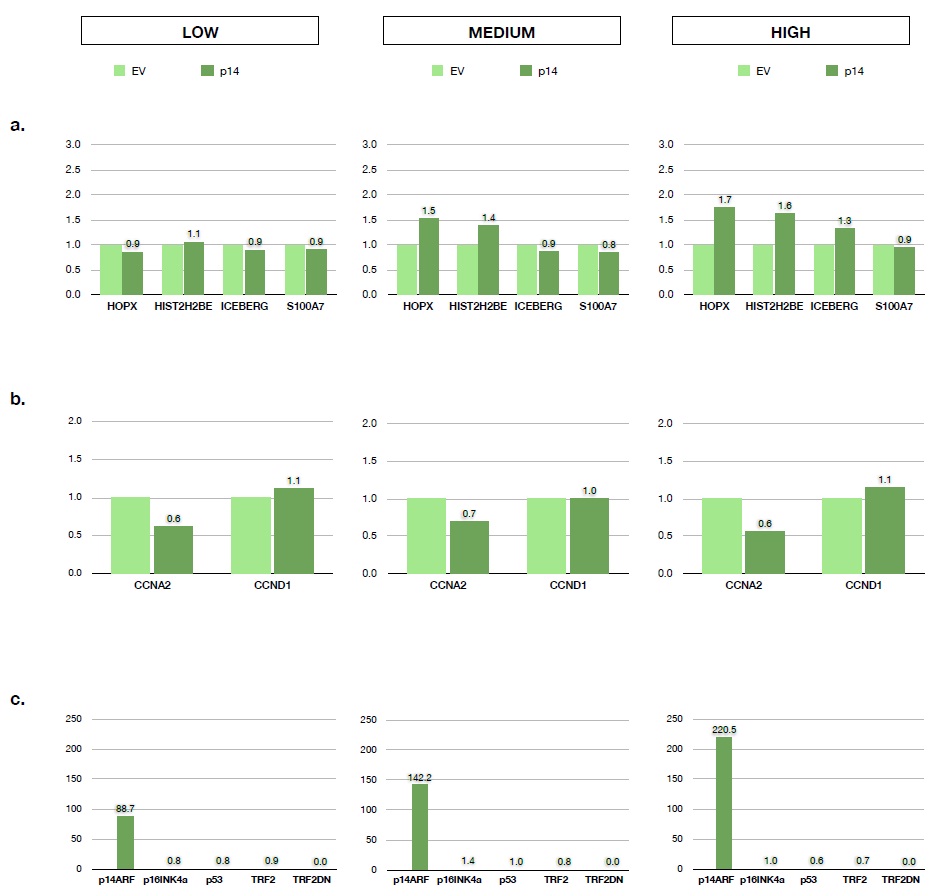

Supplement: Additional file 1: Figure S1 — Transcriptional profile of populations of normal human epidermal keratinocytes expressing p14ARF. NHEKs were transduced with p14ARF in three independent experiments resulting in keratinocyte populations expressing p14ARF at different levels: LOW (89-fold), MEDIUM (142-fold) and HIGH (220-fold). Cell extracts were analysed 5 days following expression of the transgene by RT-qPCR for induction of transcript levels of (a) HOPX, HIST2H2BE, ICEBERG and S100A7 (S100A7/S100A15); (b) Cyclin A2 (CCNA2) and Cyclin D1 (CCND1) and (c) effectors of senescence-associated cell cycle arrest p14ARF, p16INK4A and p53. Data are reported as fold increase in mRNA expression levels relative to the respective empty vector (EV) control. Legend: EV, NHEK expressing empty vector control; p14, NHEK expressing p14ARF. [file 2046-2395-3-8-S1.jpeg]

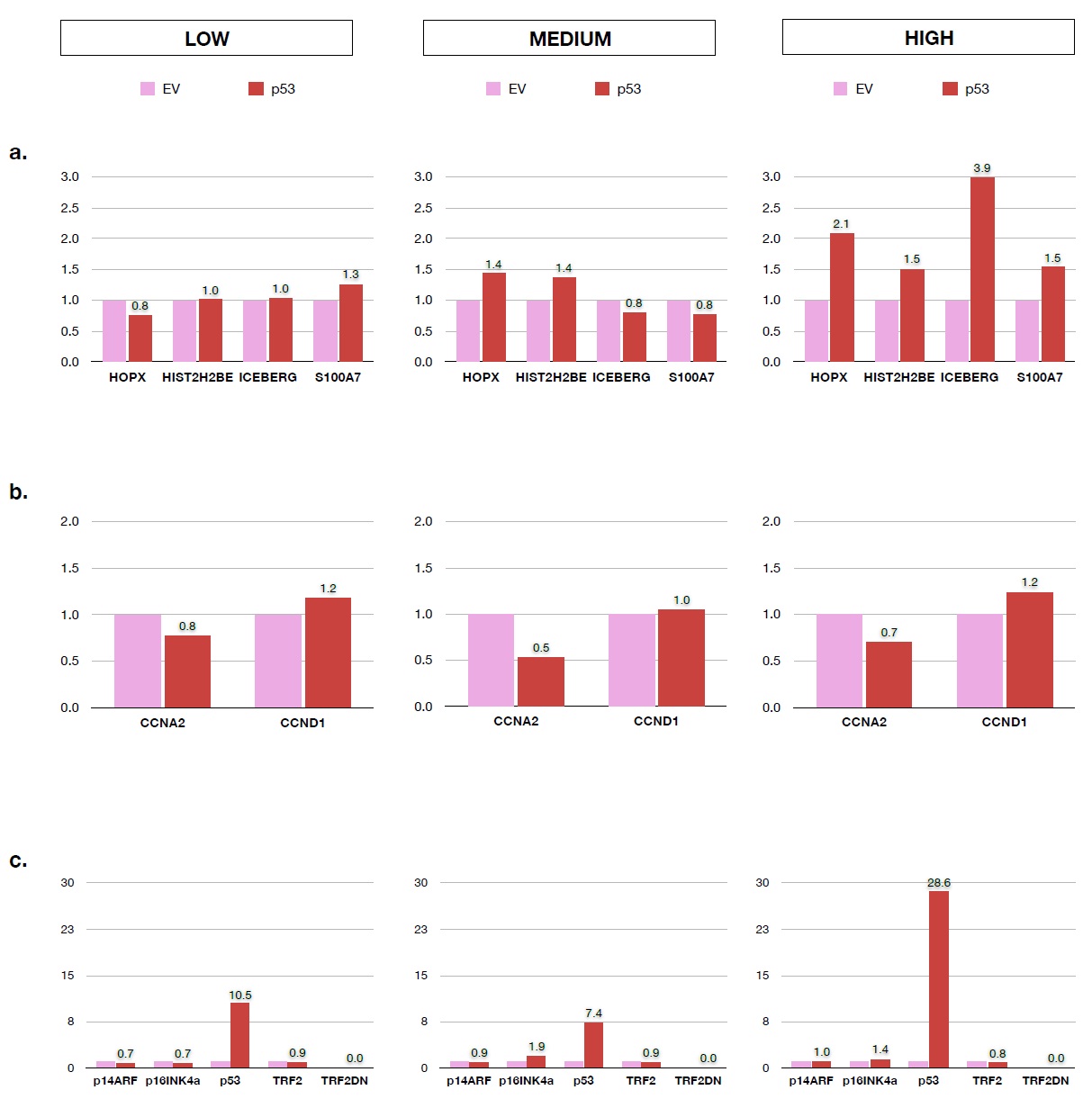

Supplement: Additional file 2: Figure S2 — Transcriptional profile of populations of normal human epidermal keratinocytes expressing p53. NHEKs were transduced with p53 in three independent experiments resulting in keratinocyte populations expressing p53 at different levels: LOW (10-fold), MEDIUM (7-fold) and HIGH (29-fold). Cell extracts were analysed 5 days following expression of the transgene by RT-qPCR for induction of transcript levels of (a) HOPX, HIST2H2BE, ICEBERG and S100A7 (S100A7/S100A15); (b) Cyclin A2 (CCNA2) and Cyclin D1 (CCND1) and (c) effectors of senescence-associated cell cycle arrest p14ARF, p16INK4A and p53. Data are reported as fold increase in mRNA expression levels relative to the respective empty vector (EV) control. Legend: EV, NHEK expressing empty vector control; p53, NHEK expressing p53. [file 2046-2395-3-8-S2.jpeg]
